# Dietary xylo-oligosaccharide supplementation alters gut microbial composition and activity in pigs according to age and dose

**DOI:** 10.1186/s13568-019-0858-6

**Published:** 2019-08-27

**Authors:** Jie Pan, Jie Yin, Kai Zhang, Peifeng Xie, Hao Ding, Xingguo Huang, Francois Blachier, Xiangfeng Kong

**Affiliations:** 1 College of Animal Science and Technology, Hunan Agriculture University, Changsha, 410128 Hunan China; 2 Key Laboratory of Agro-ecological Processes in Subtropical Region, Hunan Provincial Key Laboratory of Animal Nutritional Physiology and Metabolic Process, National Engineering Laboratory for Pollution Control and Waste Utilization in Livestock and Poultry Production, Institute of Subtropical Agriculture, Chinese Academy of Sciences, Changsha, 410125 Hunan China; 3 Nutrition Physiology and Ingestive Behavior, UMR 914 INRA/AgroParisTech/Universite Paris-Saclay, Paris, France; 4 Hunan Co-Innovation Center of Animal Production Safety, Changsha, 410128 Hunan China

**Keywords:** Growing and fattening period, Gut microbiota, Bacterial metabolites, Pigs, Feed antibiotics

## Abstract

This study explored the effect of dietary xylo-oligosaccharide (XOS) supplementation on the gut microbial composition and activity in pigs of different ages. Eighty pigs with an average body weight (BW) of 30 kg were randomly divided into eight groups: A control group, a group that received antibiotic treatment, and six groups fed diets supplemented with 100, 250, and 500 g/t XOS, of which three groups were in the growing period (GP, 30–65 kg BW) and three groups in the growing and fattening period (GFP, 30–100 kg BW). At the end of the experiment, the intestinal contents were sampled for analyses of gut microbiota and bacterial metabolites including short-chain fatty acids (SCFAs) and bioamines. The results showed that 100 g/t XOS supplementation during the GFP significantly reduced the relative abundances of presumably pathogenic bacteria (*Proteobacteria* and *Citrobacter*), but enhanced the relative abundances of likely beneficial bacteria (*Firmicutes* and *Lactobacillus*). However, XOS supplementation during the GP showed little effect on the gut microbiota when pigs were killed at 100 kg BW. Meanwhile, 100 g/t XOS supplementation during the GFP decreased the level of 1,7-heptane diamine and increased the acetic acid, straight-chain fatty acids, and total SCFAs concentrations in the intestinal contents. Statistical analysis showed that both the dose and exposure time to XOS supplementation affected the microbial communities. In summary, 100 g/t XOS supplementation during the GFP modified the gut microbiota composition and metabolic activity. Possible consequences of such changes for the host are discussed.

## Introduction

Feed antibiotics have been widely used to promote animal growth and feed conversion rates, with the serious risk of generating antibiotic-resistant bacteria and genes (Yan et al. 2018). Such risks may lead to the spread of zoonotic diseases and the presence of antibiotic residues in pork products and in the environment, which poses potential threats to human health and environmental safety (Chiesa et al. 2017). However, prohibited or restricted use of antibiotics may increase the susceptibility of pigs to endogenous or exogenous bacterial diseases (Yan et al. 2018). Thus, many recent studies have focused on developing antibiotic substitutes to improve animal growth and health without potential threats. These tested substitutes include prebiotics, bacteriophages, plant-derived phytochemicals, Chinese herbal medicine additives, antimicrobial peptides, and organic acids (Dewulf et al. 2013; Yin et al. 2018a, b).

Gut microbes form a complex system that can be affected by diet, age, and environmental factors (Wu et al. 2018). Previous studies demonstrated that prebiotics may selectively lead to an increase in the abundance of bacteria, such as *Firmicutes*, *Bifidobacterium*, and *Lactobacillus*, which are often considered as beneficial in terms of their capacity to produce short-chain fatty acids (SCFAs) (Yin et al. 2018b). These bacterial metabolites have been shown to be used as fuel by the absorptive colonic epithelial cells (Ferreira-Lazarte et al. 2018). Among the SCFAs, butyrate is involved in the regulation of the gene expression in colonocytes and is related to some anti-inflammatory effects, the maintenance of the gut barrier function, water-electrolyte balance, and several effects on intestinal metabolism (Cheng et al. 2018). Xylo-oligosaccharide (XOS), a kind of functional polymeric carbohydrate, have been widely reported to promote *Bifidobacterium* proliferation and improve host immunity (Yin et al. 2019). In broilers, XOS supplementation has been shown to increase growth performance that is associated with changes in the intestinal microbial compositions (Ribeiro et al. 2018), which suggests that such supplementation and its related changes to the intestinal ecosystem may prove to be useful for animal production. Also, Nawaz et al. (2018) found that XOS treatment improved gut health by directly affecting the epithelial barrier. However, little research has focused on the microbial responses to different doses of dietary XOS at different pig growth stages. Therefore, the present study aimed to test the hypothesis that dietary supplementation with different doses of XOS alters the gut microbiome composition and activity in different growth stages of pigs.

## Materials and methods

### Study design and sample collection

**Fig. 1:**
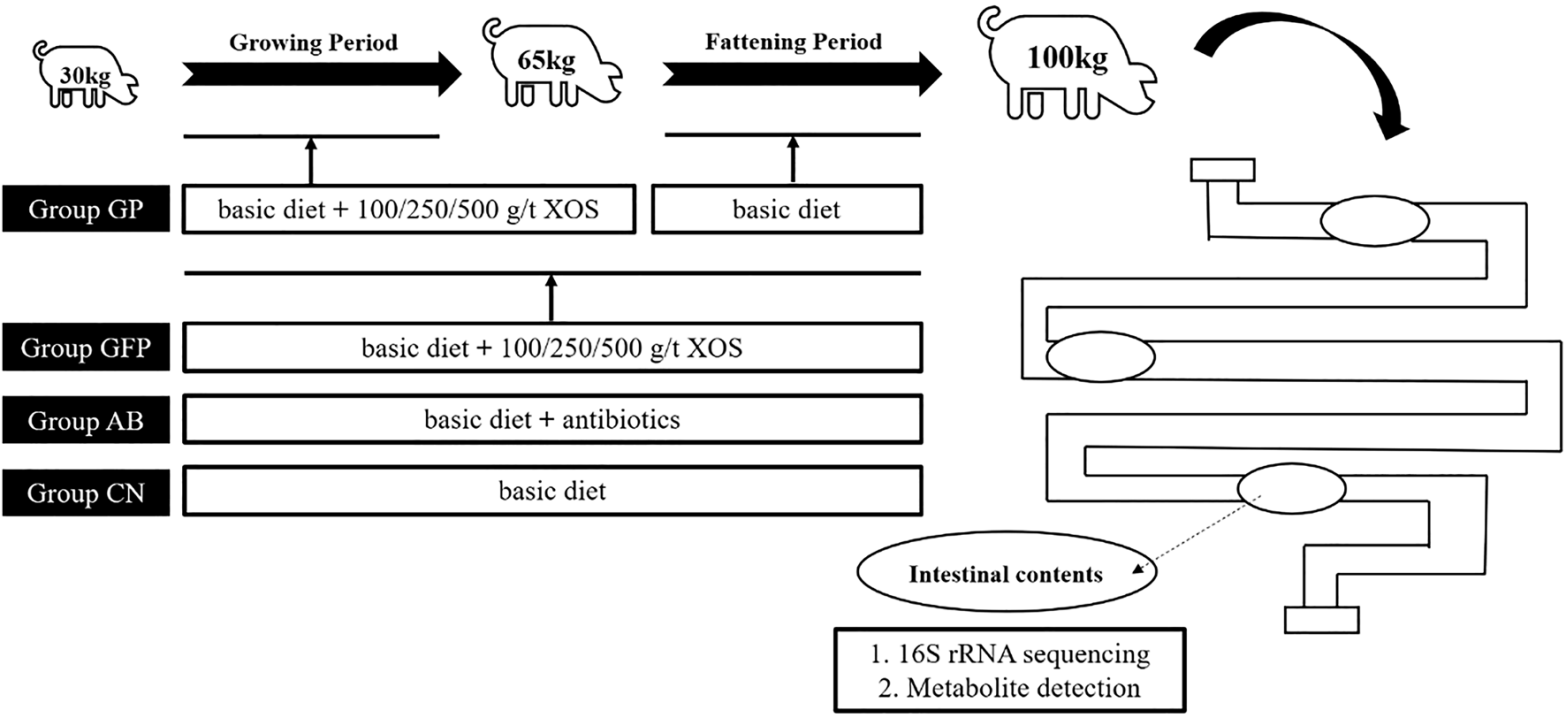
Experimental protocol

The study protocol (shown in Fig. 1) was approved by the animal welfare committee of the Institute of Subtropical Agriculture, Chinese Academy of Sciences. Eighty Duroc × Landrace × Yorkshire pigs (70 days of age) with an average body weight (BW) of ~ 30 kg were divided randomly into eight groups (n = 10, half male and half female) (Table 1). (1) CN group: pigs received a basal (control) diet (Table 2); (2) AB group: pigs received a basal diet containing antibiotics (0.04 kg/t virginiamycin and 0.2 kg/t colistin); (3–5) GP 100/250/500 groups: pigs received a basal diet with 100/250/500 g/t XOS supplementation, respectively, during the growing period (average BW: 30–65 kg) and a basal diet during the fattening period (average BW: 65–100 kg); (6–8) GFP 100/250/500 groups: pigs received a basal diet with 100/250/500 g/t XOS supplementation, respectively, during the growing-fattening period. The XOS provided by Shandong Longlive Biotechnology Co., Ltd (Shandong, China), contained xylobiose, xylotriose, and xylotetraose at ≥ 35%. The basal diet of all groups was the same and prepared according to the NRC (2012) pig nutrition requirement standard. The animal feeding experiment was conducted at the Yongan Animal Testing Base of the Institute of Subtropical Agriculture, Chinese Academy of Sciences from June to September 2015. The feeding management was strictly in accordance with the specifications for commercial farms.

**Table 1:** Experimental animal groups according to different doses of XOS in animals at different growth stages

Items	Basic diet	Antibiotics	XOS addition doses (g/t)		
			100	250	500
Growing period	CN	AB	GP 100	GP 250	GP 500
Growing-fattening period			GFP 100	GFP 250	GFP 500

**Table 2:** Composition and nutrient levels of basal diets (air-dry basis; %)

Items	Diet of growing pigs	Diet of fattening pigs
Corn	60.00	61.00
Barley	6.00	8.00
Soybean oil	2.00	1.50
Soybean meal	27.50	25.00
Calcium hydrogen phosphate	0.10	0.10
Lysine	0.16	0.18
Methionine	0.02	0.03
Threonine	0.10	0.07
Antioxidants	0.02	0.02
Mold inhibitor	0.10	0.10
Premix^a^	–	4.00
Premix^b^	4.00	–
Total	100.00	100.00
Nutrition level^c^		
Total digestion energy/(MJ/kg)	13.92	13.78
Crude protein	17.20	16.40
Crude fat	4.70	4.30
Lysine	1.17	1.08
Methionine	0.33	0.30
Threonine	0.77	0.71
Calcium	0.77	0.74
Total phosphorus	0.56	0.52

^a, b^The premix compositions were in accordance with NRC (2012) recommended nutrient requirements for growing and fattening pigs

^c^Nutrient levels were calculated values

### Quantitation of bacterial metabolites in colonic contents

When the average BW of each group of pigs reached ~ 100 kg (about 170 days of age), the feed was removed, and the pigs were slaughtered 12 h later. All pigs were transported from the farm to the processing facility (~ 40 km) at 0700 h and slaughtered at 1900 h under commercial conditions using electrical stunning (120 V, 200 Hz) and exsanguination (Hu et al. 2017). After colon recovery, the luminal contents were collected from a 10-cm region at the end of the colon and stored at − 80 °C. The colonic contents were homogenized and centrifuged at 1000*g* for 15 min, as described previously (Kong et al. 2016), before determining the metabolites.

The intestinal SCFAs, including straight-chain fatty acids (acetate, propionate, butyrate, and pentanoate) and branched-chain fatty acids (BCFA; isobutyrate and isopentanoate) were detected by gas chromatography, as described previously (Zhou et al. 2014). The bioamines, including 1,7-heptyl diamine, cadaverine, putrescine, tryptamine, tyramine, spermidine, and spermine were determined by high-performance liquid chromatography, as described previously (Xu et al. 2014).

### DNA extraction, 16S ribosomal RNA amplicon and sequencing

Microbial genomic DNA was extracted from all samples using a HiPure Stool DNA Kit (Magen, Guangzhou, China) following the manufacturer’s instructions. The final DNA concentration and purification were determined using a NanoDrop 2000 UV–vis spectrophotometer (Thermo Fisher Scientific, Waltham, MA, USA), and DNA quality was checked using 1% agarose gel electrophoresis. Illumina MiSeq sequencing and general data analyses were performed by a commercial company (Magigene, Guangzhou, China). The V4 hypervariable regions of 16S rDNA were amplified by PCR using 515F/806R primers and the following protocol (Bates et al. 2010). One pre-denaturation cycle at 94 °C for 5 min, followed by 25 cycles of denaturation at 94 °C for 30 s, annealing at 52 °C for 30 s, elongation at 72 °C for 45 s, and one post-elongation cycle at 72 °C for 10 min. PCR reactions were carried out in a 60 μL mixture containing 6 μL of 10× Ex Taq buffer, 6 μL of dNTPs, 0.6 μL of BSA, 1.2 μL of each primer, 0.3 μL of Ex Taq, 1 μL of template DNA, and 43.7 μL of ddH_2_O. The PCR amplicon products were separated on 2% agarose gels, purified, pooled at equimolar concentrations, and paired-end sequenced (2 × 250) on an Illumina MiSeq platform according to the standard methods.

### Bioinformatic analysis

High-quality sequences were uploaded to QIIME (version 1.7.0) for further study. Raw data was mass filtered using Trimmomatic software to obtain quality-controlled clean data. Each pair of PE reads was then spliced using Mothur (version 1.3) and FLASH (version 1.2.1) software, to obtain the original spliced sequence (Raw contigs). The quality of the spliced sequence was controlled and filtered by Mothur to obtain effective splicing fragments (clean contigs). The spliced sequences were then assigned to the corresponding samples by Qiime according to the barcode and primer information. Preprocessed sequence reads were clustered into operational taxonomic units (OTUs) with similarity thresholds of 97% using Usearch software (version 7.1), after which the chimera and singletons were also eliminated by the Usearch software (version 7.1). A representative sequence from each OTU was selected using QIIME. The representative sequences were then classified by species to better clarify the source of all sequence species using QIIME. QIIME was used for representative sequence species annotation. RDP Classifier was used for species annotation, and the database used was Green genes. The taxonomy results of species annotation were divided into 6 levels, namely phylum (L2), class (L3), order (L4), family (L5), and genus (L6). The analysis of the alpha diversity indexes, including Chao1, Shannon, and Simpson were calculated using QIIME. Beta-diversity analysis, including an unweighted UniFrac principal coordinate analysis (PCoA) was performed using QIIME. Least discriminant analysis effect size (LEfSe) and LDA effect size were applied to identify differences in the dominant bacterial community. To clarify the relationship between microbial community structure and environmental factors, a canonical correspondence analysis (CCA) was conducted using CANOCO software.

### Statistical analysis

Data represent the means ± SE. For metabolites (including SCFAs and bioamines), a general linear model (GLM) and least significant difference (LSD) test were used to analyze data among more than two groups using SPSS 22.0 (IBM, USA). Differences with *P* < 0.05 were considered statistically significant, while a tendency was considered to exist at 0.05 ≤ *P* < 0.10.

## Results

### Gut microbiota compositions

A total of 2,487,651 raw reads were generated from 46 samples of intestinal contents. After filtering and quality control measures, we obtained 1,805,687 clean contigs. At a threshold of 97% sequence identity, all effective reads were clustered into OTUs. For all samples, the rarefaction curves reached near plateau, which indicated that the sampling depth and sequencing coverage were appropriate for further analysis.

**Fig. 2:**
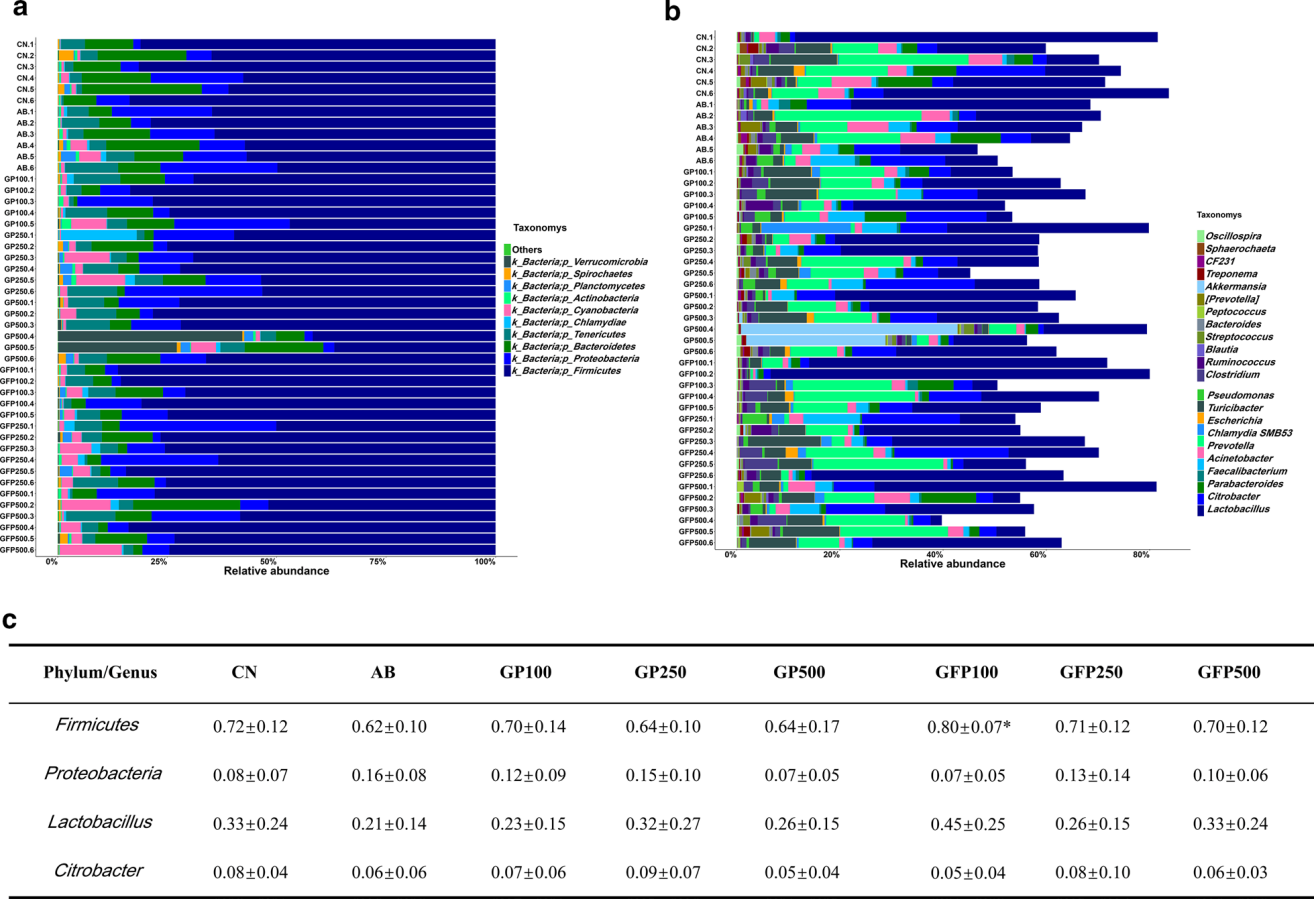
Composition of bacterial communities after XOS supplementation used at different concentrations in pigs at different stages of development. **a** Relative contribution of the top 10 phyla in each sample. **b** Relative contribution of the top 23 genera in each sample. **c** Mean relative abundance of the phyla *Firmicutes* and *Proteobacteria* and the genera *Lactobacillus* and *Citrobacter*. GFP100 group compared with antibiotic group, *P < 0.05

At the phylum level, 24 phyla were identified in the samples from the pig intestinal contents, of which *Firmicutes*, *Proteobacteria*, *Bacteroidetes*, and *Tenericutes* comprised the dominant community members (mean relative abundance > 1%) in all groups. As shown in Fig. 2a, antibiotic addition resulted in an increase in the relative abundance of *Proteobacteria* and a decrease in the relative abundance of *Firmicutes*, while XOS supplementation, during the GFP contributed to an increase in *Firmicutes* and a decrease in *Proteobacteria*. In addition, we found that the abundance of *Firmicutes* was markedly significantly different (*P *= 0.02) between the AB group and the GFP 100 group.

At the genus level, a total of 320 genera were identified, which were dominated by *Lactobacillus*, *Citrobacter*, *Prevotella*, *Acinetobacter*, and *Turicibacter*. *Lactobacillus*, the most abundant genera in all groups, displayed an increasing trend in the 100 g/t XOS supplemented group during GFP, but exhibited a decreasing trend in the AB group (Fig. 2b). Conversely, the relative abundance of *Citrobacter* increased in the AB group but decreased in the GFP 100 group.

### Microbiota diversity

**Fig. 3:**
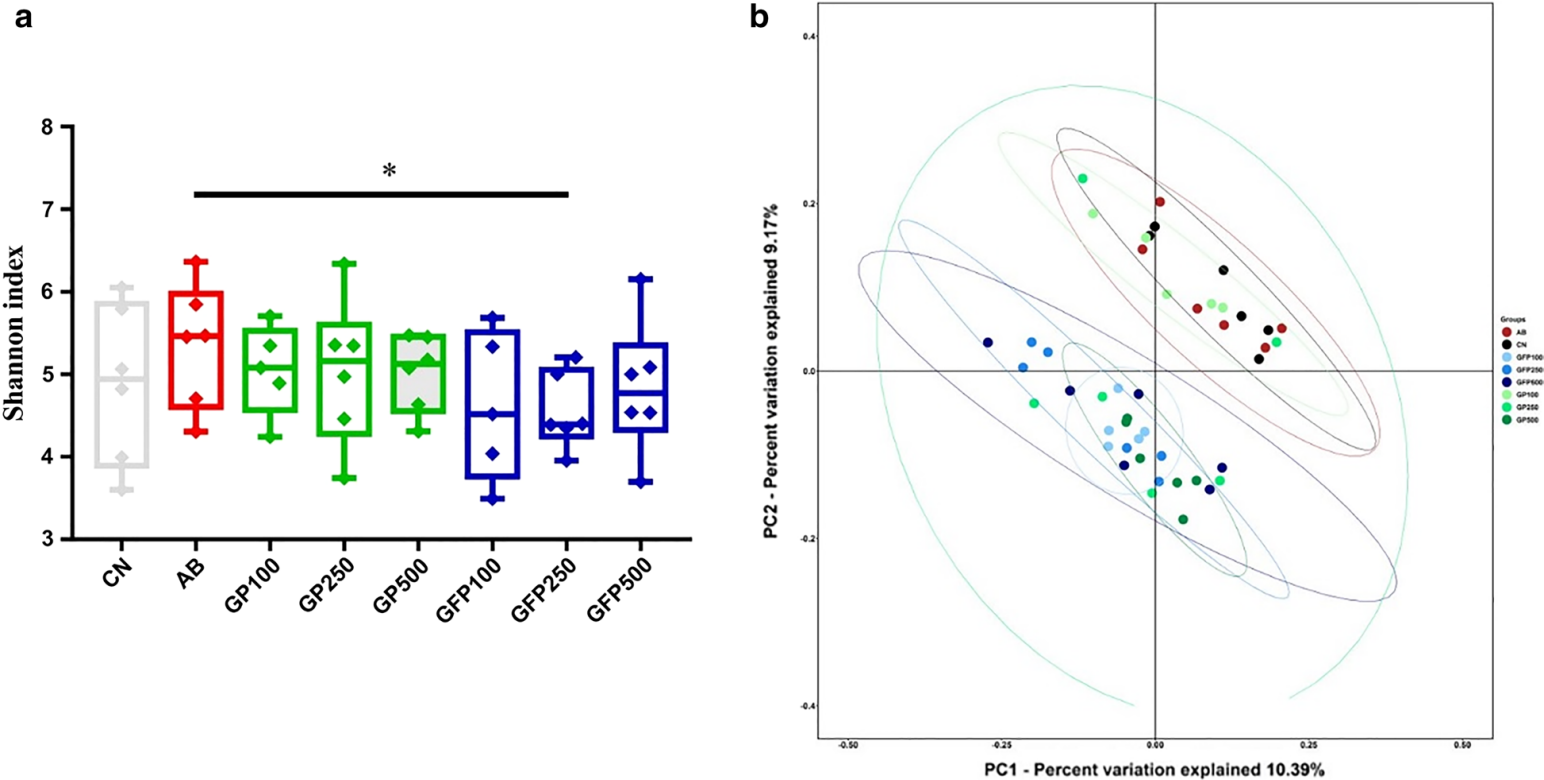
Microbial diversity in intestinal contents after supplementation with different doses of XOS in pigs at different stages of development. **a** Shannon index. **b** Scatterplot of unweighted UniFrac distance matrix Principal coordinates analysis. *P < 0.05

Several alpha diversity measures were calculated for all samples to assess the diversity, richness, and phylogenetic diversity of the bacterial community. The results clarified unambiguously that the alpha diversity values (including Observed species, Chao 1, Dominance, PD whole tree, Shannon, and Simpson indices) exhibited no significant differences between the CN and any XOS addition groups (data not shown), while significant differences in the Shannon index were observed between the GFP 250 group and AB group (*P *< 0.05) (Fig. 3a).

The PCoA based on the unweighted UniFrac distance matrix clustered all replicate intestinal content samples together within the same treatment plot and they displayed stronger dissimilarities to those from other treatments (Fig. 3b). In the GFP groups, although sample overlapping occurred among the three dose groups, clustering tendencies were observed, which exhibited a greater similarity in the gut microbiota structure between the GFP 100 group and GFP 250 group. In the GP groups, there were significant clustering tendencies in both the GP 100 group and GP 500 group, between which the tendency to stay away from each other was observed. At the same XOS supplementation dose, clustering occurred at different stages of XOS addition and they became progressively more distinct from each other. In summary, the growth stage at which XOS was added may be the major force shaping the gut microbiota structure, while dose played an insignificant role.

### Screening for microbial biomarkers

**Fig. 4:**
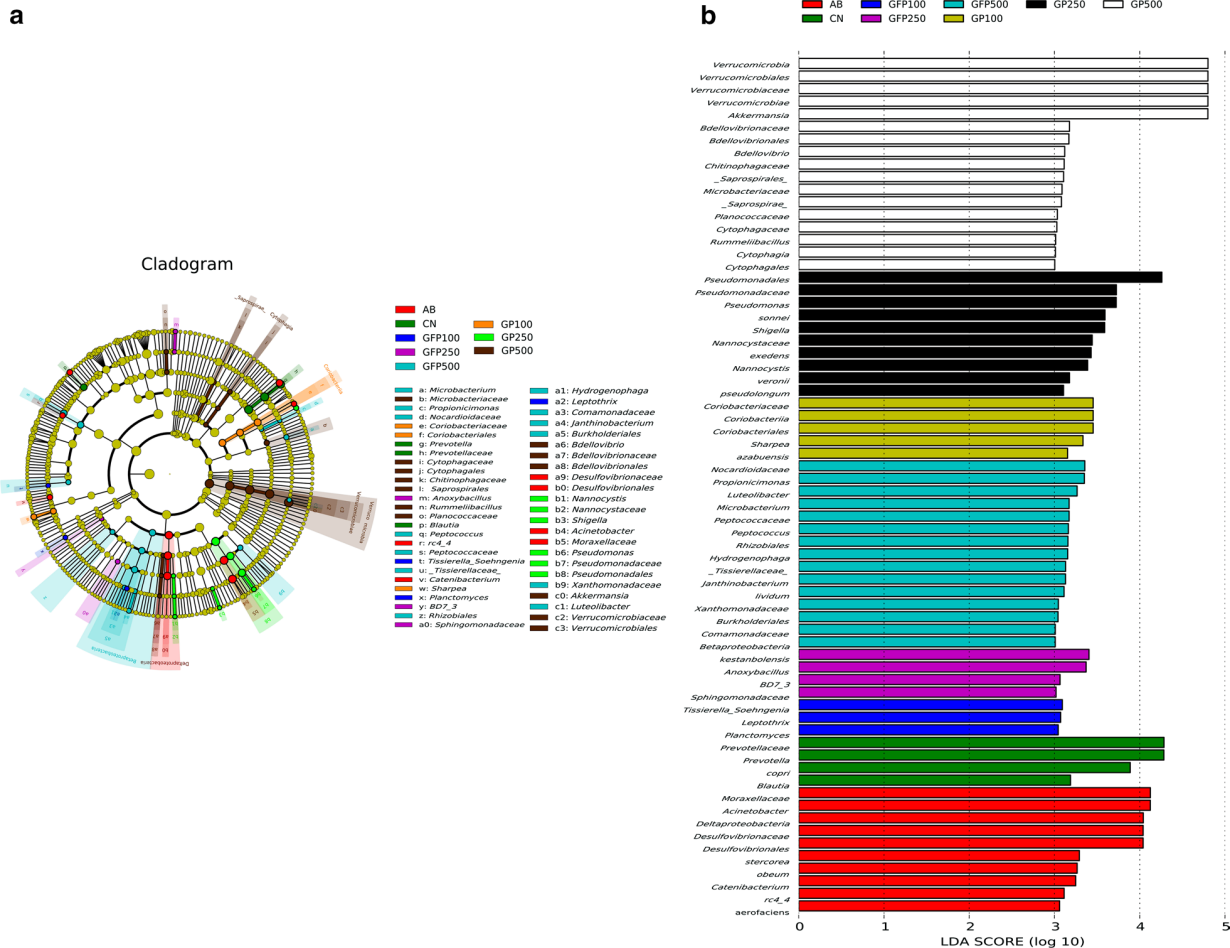
LEfSe analysis filtered out the biomarkers of the microbial community after supplementation with different doses of XOS in pigs at different stages of development. **a** Cladogram plot of LEfSe analysis. **b** Histogram of LDA analysis

The LEfSe was used to identify bacterial biomarkers that were associated with different XOS treatments (Fig. 4). In our study, the CN group was associated with the increased relative abundances of *Clostridia* and *Bacteroidia* at the class level. In contrast, the AB group had elevated levels of several classes in different phyla such as *Gammaproteobacteria* and *Deltaproteobacteria* in the *Proteobacteria*, *Clostridia*, and *Erysipelotrichi* in the *Firmicutes*, *Coriobacteriia* in the *Actinobacteria*, and *Bacteroidia* in the *Bacteroidetes*. The GP 100 group was associated with an increased relative abundance of *Erysipelotrichi* in the *Firmicutes* and *Coriobacteriia* in the *Actinobacteria*. In addition, the microbial biomarkers of these bacterial classes in the GP 250 group were thereafter identified, which showed that the abundance of *Actinobacteria*, *Gammaproteobacteria*, and *Deltaproteobacteria* were significantly higher than in the other groups, while the dominant species in the GP 500 group were predominantly from the classes *Cytophagia*, *Saprospirae*, *Verrucomicrobia*, *Bacilli*, *Actinobacteria*, and *Deltaproteobacteria*. However, *Clostridium* (*P* = 0.021) in the *Firmicutes* phylum, *Planctomyces* (*P *= 0.018) in the *Planctomycetes* phylum, and *Betaproteobacteria* (P = 0.005) in the *Proteobacteria* phylum had higher abundances in the GFP 100 group, while significant microbial biomarkers in the GFP 250 group were all derived from the phylum *Proteobacteria*, including the classes *Bacilli* and *Alphaproteobacteria*. The dominant species in the GFP 500 group were from the classes *Betaproteobacteria*, *Alphaproteobacteria*, *Gammaproteobacteria*, *Actinobacteria*, *Clostridia*, and *Verrucomicrobiae*.

### Effects of environmental factors on microbial communities

**Fig. 5:**
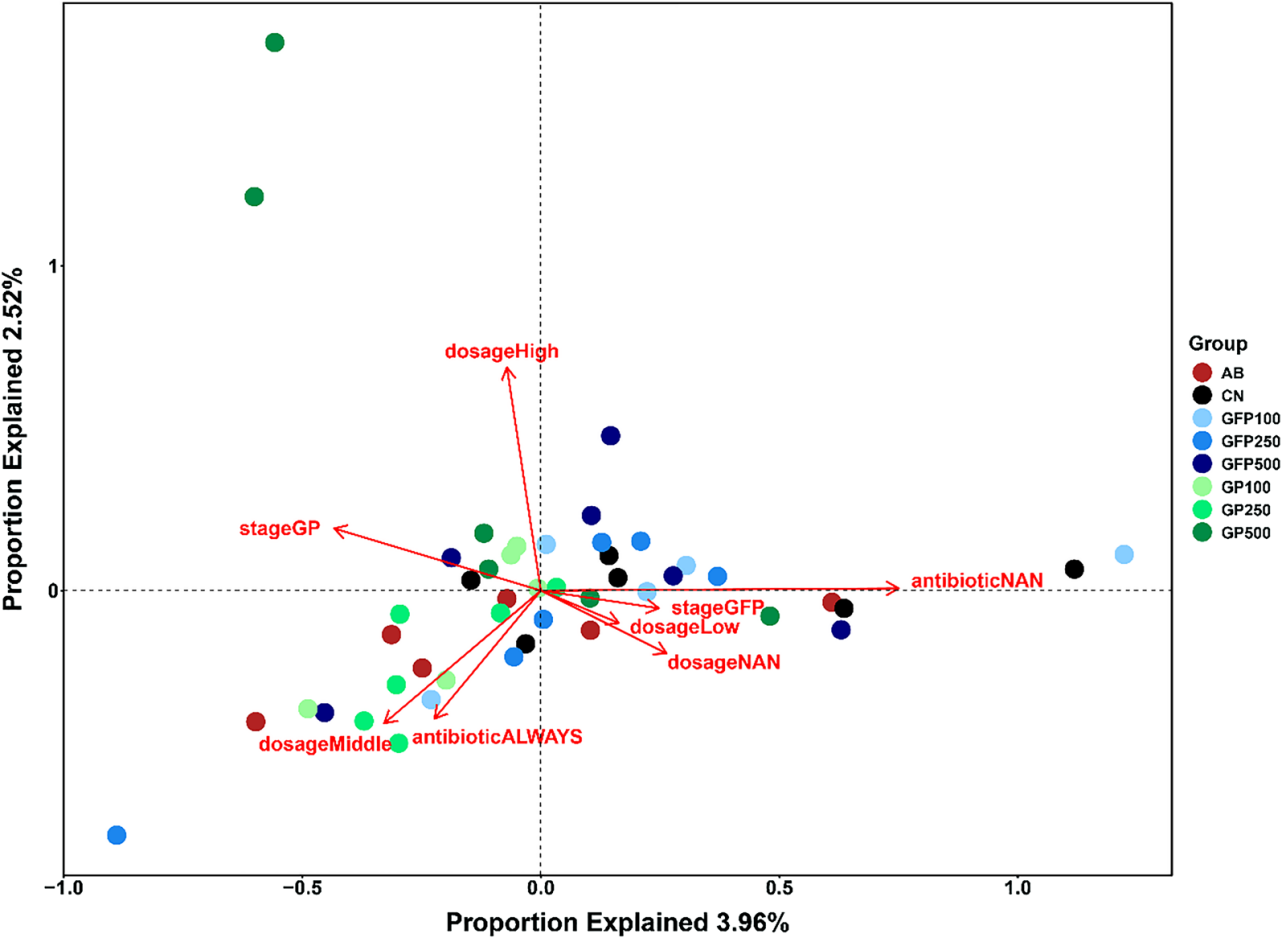
Effects of different dietary conditions on the structure of microbial communities using RDA analysis. Dose NAN: no XOS addition; dose low: 100 g/t XOS addition; dose Middle: 250 g/t XOS addition; dose high: 500 g/t XOS addition; antibiotic NAN: no antibiotics and XOS addition; antibiotic ALYWAS: antibiotic addition only; stage GP: XOS addition during growing period; stage GFP: XOS addition during growing-fattening period

An RDA analysis was performed with a permutation test to determine the correlation of different factors to the structure of the intestinal microbial community. The results of the DCA analysis performed prior to the RDA analysis showed that the axis length of the first axis was less than 3 (Fig. 5). RDA analysis results showed that both the XOS dose (including four levels: dose NAN, dose LOW, dose Middle, and dose High) (r^2^ = 0.5789, *P* < 0.001) and addition style (including four types: antibiotic NAN, antibiotic ALWAYS, stage GP, and stage GFP) (r^2^ = 0.5153, *P* < 0.001) were significantly shaped the structure of the intestinal microbial communities. Of all specific environmental factors examined, antibiotics NAN, dose High, and stage GP were significantly correlated with microbial community structure, followed by the other factors: dose Middle, antibiotic ALWAYS, and dose NAN. With regards to the correlation between various factors, the middle and low doses were negatively correlated with the high dose, while XOS addition in GFP was negatively correlated with XOS addition in GP.

### Metabolites analysis

**Fig. 6:**
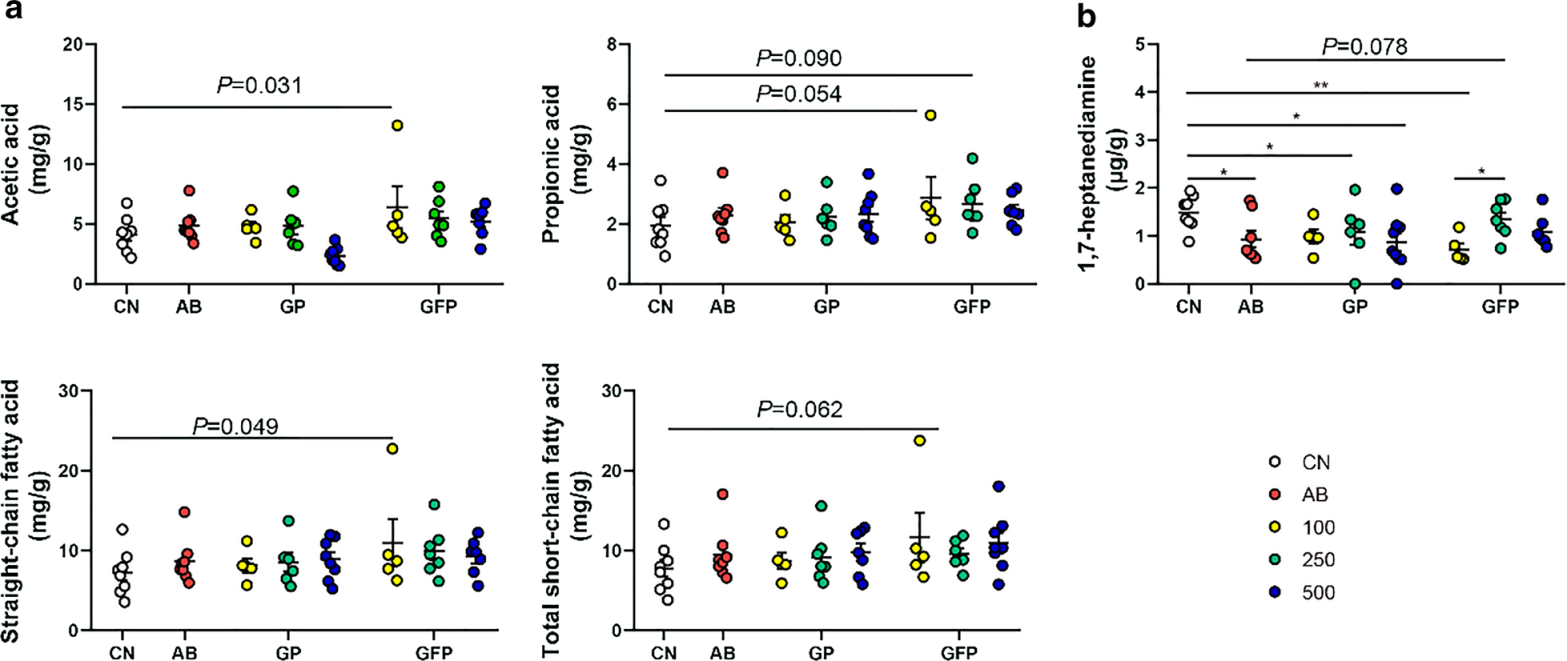
Concentrations of short-chain fatty acids (SCFA) in fresh colonic contents after supplementation with different doses of XOS in pigs at different stages of development. **a** Scatter plot of SCFA concentration. **b** Scatter plot of 1,7-heptane diamine concentration. *P < 0.05; **P < 0.01. The straight-chain fatty acids = acetate + propionate + butyrate + pentanoate; the total short-chain fatty acids = straight-chain fatty acids + isobutyrate + isopentanoate

The SCFAs were measured to assess whether XOS interventions altered the fermentation and metabolism of certain intestinal microorganisms. Compared to the basal diet, 100 g/t XOS supplementation during the GFP significantly promoted the increase in acetic acid (*P *= 0.031) and total straight-chain fatty acid contents (*P *= 0.049) (Fig. 6a), while it had little effect on other straight-chain and branched-chain fatty acids (*P *> 0.05) (Additional file 1: Table S1). In addition, the dose of XOS supplementation was not found to be correlated with the production of all types of SCFAs (*P *> 0.05). Bioamines were detected to determine the body’s amino acid metabolism. In several groups, including AB, GP 250, GP 500, and GFP 100, a significant decrease in the concentration of 1,7-heptane diamine was observed (*P *< 0.05) (Fig. 6b), especially in the GFP 100 group whereby 1,7-heptane diamine reached the lowest level, indicating that XOS had the ability to inhibit decarboxylation of amino acids. However, other bioamines has no significant effect (*P * > 0.05) (Additional file 1: Table S1).

## Discussion

Our previous study showed that dietary supplementation with different doses of XOS does not affect the growth performance of pigs but improves the pork nutritional value via increasing muscular crude protein content (Pan et al. 2017). In the present study, our data clearly showed that dietary supplementation with 100 g/t XOS during the GFP increased the relative abundance of certain bacteria, e.g., *Lactobacilli*, with presumably beneficial effects for the host, and also increased several bacterial metabolite concentrations, such as SCFAs and bioamines, in pigs.

Alpha diversity refers to diversity within a particular region or ecosystem and has been considered to be a comprehensive indicator of species richness and evenness. In the current study, the Shannon index in the GFP 250 group was significantly lower than that in the AB group, which suggested that dietary supplementation with 250 g/t XOS during GFP can significantly decrease the diversity of the intestinal microbiota. Similarly, Yang et al. (2018) reported that XOS intervention in mice with chronic kidney disease caused a significant decrease in the alpha diversity of the fecal microbiota, evidenced by a decrease in the relative abundances of six major genera. Beta diversity refers to the heterogeneity of species composition between different habitat communities along the environmental gradient, which reflects the species diversity between communities. The current study demonstrated that XOS addition significantly altered the beta diversity of microorganisms.

Generally, *Firmicutes*, *Bacteroides*, *Proteobacteria*, and *Fusobacterium* occupy the dominant phyla of mammalian guts (Guevarra et al. 2018). In the current study, *Firmicutes*, *Proteobacteria*, *Bacteroides*, and *Tenericutes* were the dominant phyla after dietary XOS intervention for about 6 months in pigs. XOS has been widely demonstrated to selectively promote *Bifidobacterium* counts, while there is still controversy regarding the changes in *Lactobacilli* (Finegold et al. 2014). Numerous studies have shown that *Lactobacillus* is closely related to the production of SCFAs, which, as organic acids, can lower the pH in the intestine, promote gastrointestinal motility, and inhibit the growth and reproduction of nitrate-reducing bacteria (Makelainen et al. 2010; Tana et al. 2010). In the present study, the levels of acetic acid, propionic acid, and straight-chain fatty acids in the GFP 100 group were significantly higher than those in the CN group, which suggests that certain related bacteria, such as *Lactobacilli*, may play key roles in SCFA production. Indeed, the relative abundance of *Lactobacillus* in the GFP 100 group was higher compared with the other groups. Similarly, Ding et al. (2018) reported that the acetic acid content in the intestinal tract of chicken increased linearly with the increase in XOS concentration in the diet.

SCFAs are microbiota products of the fermentation of carbohydrates and amino acids, while bioamines are produced from amino acids (as released from luminal protein) by intestinal bacteria (Johansson et al. 1998), notably by various bacteria such as *E. coli* that play an auxiliary role (Blachier et al. 2007). Bioamines (i.e., 1,7-heptyl diamine, cadaverine, putrescine, tryptamine, tyramine, spermidine, and spermine) are markers of potentially harmful metabolic pathways. However, the polyamine putrescine is well known to be involved in the epithelial cell proliferation and differentiation, and thus in the epithelial renewal process (Davila et al. 2013). Results showed that the AB group, GP 250 group, GP 500 group, and GFP 100 group displayed significant decreases in the level of 1,7-heptane diamine in the intestinal contents compared with the CN group. This result, which is consistent with the relative abundance of *E. coli* in these groups, suggests that bioamine production may be related to the proliferation of *E. coli*. Compared with the CN group, the AB group reduced *Corynebacterium* abundance and 1-7 heptane diamine concentration. It is, thus, tempting to propose that antibiotics may play a critical role in the inhibition of pathogenic bacteria growth. Compared with the CN group, the AB group reduced *Corynebacterium* abundance and 1-7 heptane diamine concentration. It is thus tempting to propose that antibiotics may play a critical role in the inhibition of pathogenic bacterial growth. Additional experiments are needed to validate this proposition. In addition, compared with the AB group, XOS supplementation significantly improved the relative abundances of bacteria often considered to be beneficial, such as *Lactobacillus*, *Ruminococcus*, *Coprococcus*, and *Roseburia*; and also increased the luminal concentrations of SCFAs, which are considered to be beneficial for gut health (Azad et al. 2018).

This study explored the effect of dietary xylo-oligosaccharide (XOS) supplementation on the gut microbial composition and activity in pigs of different ages. In summary, 100 g/t XOS supplementation during the GFP modified gut microbiota composition and metabolic activity.

## Supplementary information

**Additional file 1: Table S1.** Concentrations of indole and skatole, short-chain fatty acids and bioamines in the colonic contents of pig after supplementation with different doses of XOS in pigs at different stages of development.

## Data Availability

All sequences analyzed in this study can be accessed in the SRA database under the accession number PRJNA551340 (https://dataview.ncbi.nlm.nih.gov/object/PRJNA551340).
