# Supplementary material for: Dietary xylo-oligosaccharide supplementation alters gut microbial composition and activity in pigs according to age and dose
Source: AMB Express. 2019 Aug 27;9:134. doi: 10.1186/s13568-019-0858-6 (PMC6712192; doi:10.1186/s13568-019-0858-6)
Supplement: Supplementary file 1 — Additional file 1: Table S1. Concentrations of indole and skatole, short-chain fatty acids and bioamines in the colonic contents of pig after supplementation with different doses of XOS in pigs at different stages of development. [file 13568_2019_858_MOESM1_ESM.docx]

Table S1. Concentrations of indole, skatole, short-chain fatty acids (SCFA), and bioamines in the colonic contents after supplementation with different doses of xylo-oligosaccharide (XOS) in pigs at different stages of development

| **Items** | **CN** | **AB** | **GP** | | | **GFP** | | | **SEM** | ***P* value** |
| --- | --- | --- | --- | --- | --- | --- | --- | --- | --- | --- |
|  |  |  | **100** | **250** | **500** | **100** | **250** | **500** |  |  |
| Indole (μg/g) | 10.94 | 16.41 | 10.81 | 21.54 | 9.71 | 9.11 | 8.30 | 14.94 | 3.28 | 0.10 |
| Skatole (μg/g) | 13.00 | 15.30 | 9.87 | 13.29 | 11.00 | 13.48 | 15.30 | 11.28 | 3.53 | 0.95 |
| SCFA (mg/g) |  | | | | | | | | | |
| Acetate | 4.13 | 4.85 | 4.73 | 4.82 | 5.08 | 6.38 | 5.45 | 5.20 | 0.69 | 0.54 |
| Propionate | 1.94 | 2.29 | 2.05 | 2.24 | 2.34 | 2.86 | 2.67 | 2.45 | 0.32 | 0.55 |
| Butyrate | 0.90 | 1.15 | 1.06 | 1.07 | 1.15 | 1.36 | 1.38 | 1.16 | 0.22 | 0.80 |
| Isobutyrate | 0.31 | 0.51 | 0.34 | 0.51 | 0.39 | 0.40 | 0.52 | 0.43 | 0.12 | 0.83 |
| Valerate | 0.29 | 0.39 | 0.29 | 0.41 | 0.35 | 0.36 | 0.43 | 0.43 | 0.07 | 0.77 |
| Isovalerate | 0.21 | 0.32 | 0.22 | 0.31 | 0.25 | 0.27 | 0.33 | 0.28 | 0.06 | 0.80 |
| BCFA | 0.52 | 0.82 | 0.57 | 0.82 | 0.64 | 0.66 | 0.84 | 0.71 | 0.18 | 0.82 |
| Straight-chain fatty acid | 7.26 | 8.68 | 8.13 | 8.53 | 8.92 | 10.97 | 9.94 | 9.23 | 1.25 | 0.62 |
| Total SCFA | 7.77 | 9.50 | 8.70 | 9.35 | 9.56 | 11.64 | 10.78 | 9.94 | 1.38 | 0.66 |
| Bioamines (μg/g) |  | | | | | | | | | |
| Cadaverine | 46.99 | 26.41 | 12.29 | 79.01 | 22.54 | 57.56 | 114.18 | 37.61 | 27.02 | 0.17 |
| Putrescine | 17.88 | 18.36 | 11.86 | 47.69 | 15.81 | 30.22 | 32.02 | 21.78 | 9.88 | 0.27 |
| Spermidine | 19.53 | 21.71 | 17.43 | 21.75 | 18.25 | 21.77 | 21.36 | 19.25 | 3.10 | 0.95 |
| Spermine | 2.57 | 2.57 | 1.56 | 3.63 | 2.43 | 3.00 | 2.69 | 2.71 | 0.63 | 0.67 |
| Tryptamine | 2.13 | 2.73 | 2.48 | 5.50 | 2.03 | 4.10 | 4.74 | 3.81 | 1.21 | 0.33 |
| Tyramine | 17.83 | 31.31 | 24.09 | 28.18 | 24.55 | 27.11 | 36.12 | 26.89 | 8.60 | 0.87 |
| 1,7-Heptanediamine | 1.49 | 0.93 | 0.99 | 1.08 | 0.87 | 0.72 | 1.35 | 1.09 | 0.17 | 0.49 |

BCFA: branched-chain fatty acids, including isobutyrate and isovalerate; straight-chain fatty acid, including acetate, propionate, butyrate, and valerate.
